# Serum Peptide Immunoglobulin G Autoantibody Response in Patients with Different Central Nervous System Inflammatory Demyelinating Disorders

**DOI:** 10.3390/diagnostics11081339

**Published:** 2021-07-26

**Authors:** Hye Lim Lee, Jin-Woo Park, Jin Myoung Seok, Mi Young Jeon, Hojin Kim, Young-Min Lim, Ha Young Shin, Sa-Yoon Kang, Oh-Hyun Kwon, Sang-Soo Lee, Hung Youl Seok, Ju-Hong Min, Sung-Hyun Lee, Byung-Jo Kim, Byoung Joon Kim

**Affiliations:** 1 Department of Neurology, Korea University College of Medicine, Seoul 02841, Korea; raim.hllee@gmail.com (H.L.L.); parkzinu@korea.ac.kr (J.-W.P.); 2 Department of Neurology, Soonchunhyang University Cheonan Hospital, Cheonan 31151, Korea; seok.jm@gmail.com; 3 Samsung Research Institute of Future Medicine, Seoul 06351, Korea; sarah.jeon@sbri.co.kr; 4 Department of Neurology, National Cancer Center, Goyang 10408, Korea; hojinkim@ncc.re.kr; 5 Department of Neurology, Asan Medical Center, University of Ulsan College of Medicine, Seoul 05505, Korea; limy@amc.seoul.kr; 6 Department of Neurology, Yonsei University College of Medicine, Seoul 03722, Korea; hayshin@yuhs.ac; 7 Department of Neurology, College of Medicine, Cheju National University, Cheju 63241, Korea; neurokang@cheju.ac.kr; 8 Department of Neurology, Eulji University College of Medicine, Seoul 01830, Korea; ohkwon12@gmail.com; 9 Department of Neurology, Chungbuk National University College of Medicine, Cheongju 28644, Korea; pnsdoctor@gmail.com (S.-S.L.); sunghlee@chungbuk.ac.kr (S.-H.L.); 10 Department of Neurology, Keimyung University School of Medicine, Daegu 41931, Korea; shy2354@gmail.com; 11 Department of Neurology, Samsung Medical Center, Sungkyunkwan University School of Medicine, Seoul 06351, Korea; juhong.min@samsung.com; 12 Neuroscience Center, Samsung Medical Center, Seoul 06351, Korea; 13 BK21 FOUR Program in Learning Health Systems, Korea University, Seoul 02841, Korea

**Keywords:** CNS inflammatory demyelinating disorder, peptide microarray, IgG response

## Abstract

Previous efforts to discover new surrogate markers for the central nervous system (CNS) inflammatory demyelinating disorders have shown inconsistent results; moreover, supporting evidence is scarce. The present study investigated the IgG autoantibody responses to various viral and autoantibodies-related peptides proposed to be related to CNS inflammatory demyelinating disorders using the peptide microarray method. We customized a peptide microarray containing more than 2440 immobilized peptides representing human and viral autoantigens. Using this, we tested the sera of patients with neuromyelitis optica spectrum disorders (NMOSD seropositive, *n* = 6; NMOSD seronegative, *n* = 5), multiple sclerosis (MS, *n* = 5), and myelin-oligodendrocyte glycoprotein antibody-associated disease (MOGAD, *n* = 6), as well as healthy controls (HC, *n* = 5) and compared various peptide immunoglobulin G (IgG) responses between the groups. Among the statistically significant peptides based on the pairwise comparisons of IgG responses in each disease group to HC, cytomegalovirus (CMV)-related peptides were most clearly distinguishable among the study groups. In particular, the most significant differences in IgG response were observed for HC vs. MS and HC vs. seronegative NMOSD (*p* = 0.064). Relatively higher IgG responses to CMV-related peptides were observed in patients with MS and NMOSD based on analysis of the customized peptide microarray.

## 1. Introduction

The cause of CNS inflammatory demyelinating disorders is unclear and their differential diagnosis has been challenging [1]. Therefore, numerous efforts have been made to identify new surrogate markers to differentiate CNS inflammatory demyelinating disorders. Following the identification of anti-aquaporin 4 antibody (AQP4 Ab) in patients with characteristic clinical findings, diagnostic criteria for seropositive NMOSD and seronegative NMOSD have been established and updated based on the results of many studies [2,3,4]. Research have suggested that seropositive NMOSD were associated with astrocytopathy which is pathologically distinct from classical demyelinating diseases [5]. Recently, anti-myelin oligodendrocyte glycoprotein (MOG) antibody has been identified in a few patients with clinical signs of NMOSD without AQP4 Ab [6,7,8,9].

Emerging biomarker candidates such as cytomegalovirus (CMV), human herpes virus-6 (HHV-6), varicella-zoster virus (VZV), Epstein-Barr Virus (EBV), myelin basic protein (MBP), and hypoxia-inducible factor 1 (HIF-1), have been proposed to discriminate CNS inflammatory demyelinating disorders [8,9,10,11]. Although their precise pathophysiologic mechanisms and cause-effect relationships remain unclear, they might contribute to these disease manifestations via diverse mechanisms, the evidence for which are accumulating. The role of viral antibodies has been postulated by several hypotheses such as the molecular mimicry theory [8], epitope spreading theory [12], and stimulation of autoreactive T cells by virus-encoded superantigens [13]. MBP has been suggested as a key protein in neuronal myelin sheath structure related to autoimmune-related pathogenesis [14]. The potential role of HIF-1 in infectious and inflammatory immune responses has also been suggested [15].

Therefore, the present study investigated the IgG autoantibody responses to various viral and autoantibodies-related peptides potentially related to CNS inflammatory demyelinating disorders (i.e., MS, seropositive NMOSD, seronegative NMOSD, and MOGAD) using a peptide microarray method.

## 2. Materials and Methods

### 2.1. Participants and Samples

Participant enrollment was based on the Korean Nationwide Registry for CNS demyelinating diseases (MS-NMO NETWORK), in which neurologists from major referral hospitals for neuroinflammatory and demyelinating diseases in Korea participate [16]. Among the subjects in the registry, we selected all patients who were diagnosed with MOGAD with the same positive results for MOG-IgG1 at two different laboratories. Then, patients from other groups were randomly selected by matching age, sex ratio, and expanded disability status score (EDSS) to obtain similar group sizes.

The diagnosis of MS was confirmed according to the revised McDonald criteria [17]. Cell-based assays for AQP4 and MOG Ab seropositivities were used for the accurate diagnosis of NMOSD and MOGAD. The participant groups were age-matched, with an average of 33–39 years. Considering the female predominance of each disease, the sex ratio was adjusted for the disease control groups. The EDSS was obtained at the time of blood sampling. For the possible effect of treatment on antibodies, medications that were used at the time of blood sampling were recorded. Patients receiving treatment with high-dose corticosteroids and plasma exchange within 2 months before blood sampling were excluded from the study. Patients who were pregnant or had a history of intercurrent infections at the time of blood draw were also excluded. Samples were stored at −80 °C before further analysis. Longitudinal medical records over 3 years were thoroughly reviewed, and the diagnoses were verified by expert members of MS-NMO NETWORK from the initial attack of the disease. A total of five to six patients were included in each group (NMOSD seropositive, *n* = 6; NMOSD seronegative, *n* = 5; MS, *n* = 5; MOGAD, *n* = 6; HC, *n* = 5). Our Institutional Review Board approved the study (no. 2018GR0294), and all participants provided written informed consent. All procedures were conducted in accordance with the principles described in the Declaration of Helsinki and Good Clinical Practice guidelines.

### 2.2. Peptide Microarray Design

A peptide microarray method was proposed to compare the IgG response to peptides among seropositive NMOSD, seronegative NMOSD, MS, MOGAD, and HC. We customized this method to evaluate 2440 immobilized peptides representing human and viral autoantigens potentially associated with CNS inflammatory demyelinating disorders in previous studies [18,19,20,21,22,23]. We included peptides within a length of 15 amino acids and 14 amino acid overlap from among 32 proteins from viral antigens such as CMV, HSV, EBV, and VZV and autoantigens such as MBP, HIF-1, and myelin-associated glycoprotein (MAG).

### 2.3. Microarray Staining and Reading

The 2440 selected peptides were printed in duplicate and translated into a peptide microarray (PEPperMAP^®^, Heidelberg, Germany). Pre-staining of a peptide microarray copy was done with the secondary (Goat anti-human IgG (Fc) DyLight680, Rockland Immunochemicals Inc., Limerick, PA, USA) and control antibodies (Mouse monoclonal anti-HA (12CA5) DyLight680, Rockland Immunochemicals Inc., Limerick, PA, USA) in incubation buffer (washing buffer with 10% blocking buffer, Rockland Immunochemicals Inc., Limerick, PA, USA) to investigate background interactions with the 2440 different peptides that could interfere with the main assays. Subsequent incubation (30 min) of other peptide microarray copies with the human serum samples at dilutions of 1:500 in incubation buffer was followed by staining with secondary and control antibodies (45 min) as well as read-out at scanning intensities of 7/7 (red/green). The additional HA peptides framing the peptide microarrays were simultaneously stained with the control antibody as internal quality control to confirm the assay quality and the peptide microarray integrity.

Quantification of spot intensities and peptide annotation was based on the 16-bit gray scale tiff files. Microarray image analysis was done with PepSlide^®^ Analyzer (PEPperPRINT, Heidelberg, Germany). A software algorithm breaks down fluorescence intensities of each spot into raw, foreground, and background signals, and calculates averaged median foreground intensities and spot-to-spot deviations of spot duplicates. Based on averaged median foreground intensities, intensity maps were generated and interactions were highlighted by an intensity color code with red for high and white for low spot intensities. We tolerated a maximum spot-to-spot deviation of 40%, otherwise, the corresponding intensity value (below 100 fluorescence units) was zeroed. We further plotted averaged spot intensities of the assays with the human serum samples against the peptides and antigens from left on top to right on the bottom of the microarray to visualize overall spot intensities and signal-to-noise ratios. The intensity plots were correlated with peptide and intensity maps as well as with visual inspection of the microarray scans to identify the epitopes and peptide interactions of the human serum samples.

### 2.4. Statistical Analysis

**Figure 1:**
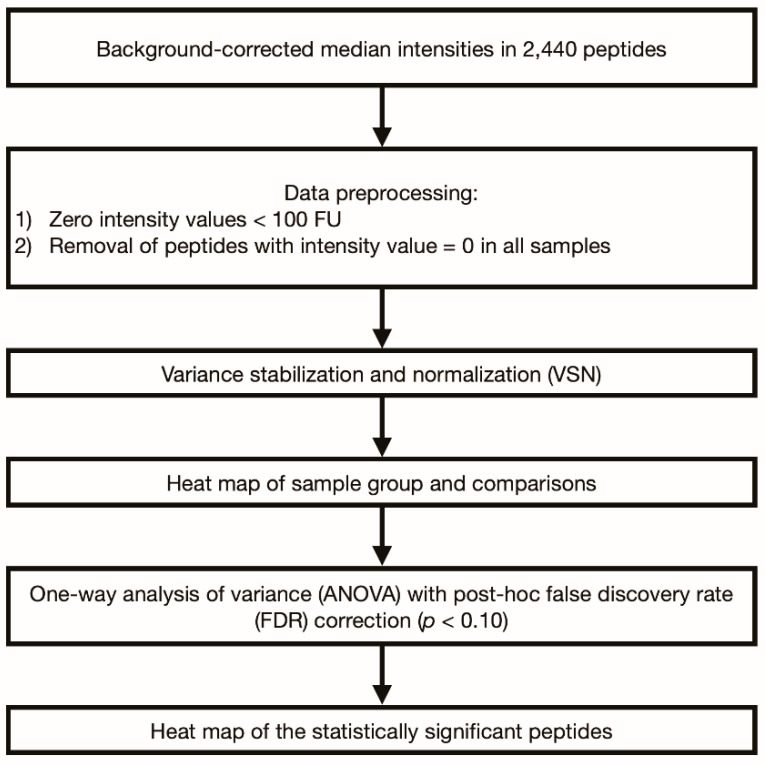
Diagram of the statistical analysis. The analysis was based on the background-corrected median intensities of IgG responses. In a pre-processing of the microarray data, all intensity values < 100 fluorescence units (FU) were zeroed, followed by removal of peptides with intensity values = 0 in all samples; this resulted in 1252 remaining peptides included in the statistical data analyses.

The schematic of the statistical analysis is described in Figure 1. The analysis was based on the background-corrected median IgG response intensities. Data processing was performed using the R language (R version 3.6.1). One-way analysis of variance (ANOVA) was used for multiple comparisons of IgG responses in each disease group and the HC group. In addition, we performed pairwise group comparisons of the IgG responses among disease groups (MS vs. seropositive NMOSD, MS vs. seronegative NMOSD, MS vs. MOGAD, seropositive NMOSD vs. seronegative NMOSD, seropositive NMOSD vs. MOGAD, and seronegative NMOSD vs. MOGAD). After data pre-processing, the samples were clustered using the Euclidean distance method. One-way ANOVA was used for multiple comparisons with Benjamini–Hochberg false discovery rate (FDR) adjustment. Statistical significance was defined as *p* < 0.10. A heatmap of statistically significant peptides were generated after variance-stabilizing normalization (VSN, vsn package, R version 3.6.1) [24].

## 3. Results

### 3.1. Participant Demographics and Clinical Data

Among the subjects in the registry, only six patients were positive for MOG-IgG at two different laboratories. By random selection with adjustment for demographics in the other groups, 22 participants were finally enrolled, including six seropositive NMOSD patients, five seronegative NMOSD patients, five MS patients, the six MOGAD patients, and five HCs. The average age was 37.8–39.3 years across the disease groups and 33.8 years in the HC group. The EDSS score did not differ significantly among the disease groups. The demographic and clinical data of the participants are presented in Table 1.

### 3.2. Analysis of IgG Autoantibody Responses of Each Disease Group Compared to the HC Group

**Figure 2:**
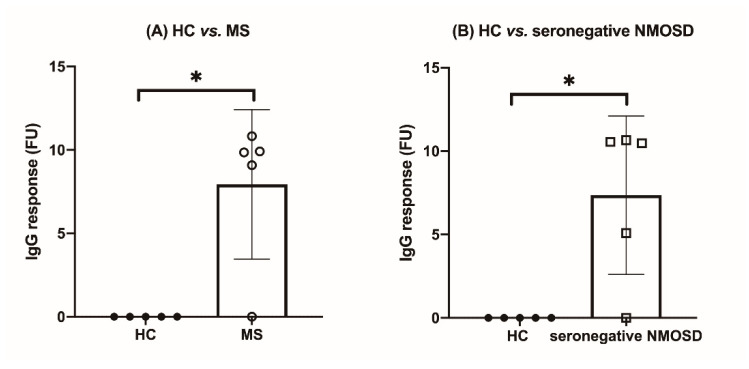
The two most IgG response differences between the study groups (* *p* = 0.064) were observed for the CMV envelope glycoprotein B-related peptide (GVNETIYNTTLKYGD) (**A**): HC vs. MS, (**B**): HC vs. seronegative NMOSD).

The IgG responses to peptides for CMV envelope glycoprotein B were significantly higher in the MS group than those in the HC group (mean difference 6.9–7.94 FU, Table 2, Supplementary Table S1). Similarly, the difference in IgG response between the seronegative NMOSD and HC groups was significant for CMV envelope glycoprotein B-related peptides (mean difference 6.28–7.35 FU, Table 2, Supplementary Table S1). These two comparisons (MS vs. HC and seronegative NMOSD vs. HC, Figure 2) were the most discriminative compared to other pairwise comparisons between the disease and HC groups.

In the seropositive NMOSD group, 24 peptides for CMV envelope glycoprotein B, CMV pp65, HIF-1 alpha, HSV glycoprotein C, and MAG showed significantly higher IgG responses than those in the HC group (Table 2, Supplementary Table S1). The highest mean difference between IgG responses to peptides was observed for the CMV pp65-related peptide (SDEELVTTERKTPRV, mean difference: 4.52 FU, *p* = 0.089). For the other peptides, the differences in IgG responses ranged from 1.6 to 2.73 FU.

No peptide showed significant differences for the comparisons between the MOGAD and HC groups.

### 3.3. Analysis of IgG Autoantibody Responses among the Disease Groups

#### 3.3.1. Comparison between MS and Seropositive NMOSD

We observed statistically significant differences in IgG responses to the 24 peptides between the MS and seropositive NMOSD groups (Supplementary Table S2). These included CMV pp65, HSV glycoprotein C, HIF-1 alpha, MAG, and CMV envelope glycoprotein B antigens. The peptide with the highest IgG response difference was a peptide of CMV pp65 (SDEELVTTERKTPRV, mean difference: 4.52 FU, *p* = 0.089, Table 2). The mean differences in IgG responses for other peptides ranged from 1.60 to 2.73 FU.

#### 3.3.2. Comparison between MS and Seronegative NMOSD

Only one peptide (GAGGAGAGGAGAGGG) for antigen type EBV-EBNA1 showed a borderline significant difference in IgG responses between the MS and seronegative NMOSD groups (5.52 FU, *p* = 0.092, data not shown).

#### 3.3.3. Comparison between Seropositive NMOSD and Seronegative NMOSD

Twenty-three peptides for CMV envelope glycoprotein B, CMV pp65, HSV glycoprotein C, HIF-1alpha, and MAG peptides showed significant differences (Supplementary Table S3), with the greatest difference in IgG response observed for CMV envelope glycoprotein B (1.60 FU, *p* = 0.087; Table 2).

#### 3.3.4. Comparison between Each Disease Group and MOGAD

Comparison between seropositive NMOSD and MOGAD also showed a similar statistically significant repertoire of peptides to those observed in the comparison between seropositive NMOSD and seronegative NMOSD (Supplementary Table S4). These peptides included those for CMV pp65, HSV glycoprotein C, HIF-1 alpha, MAG, HSV glycoprotein C, and CMV envelope glycoprotein B (mean difference ranging from −4.52 to −1.60).

However, there were no statistically significant differences in peptides in MS and seronegative NMOSD compared to those in the MOGAD group.

### 3.4. Heatmap of IgG Autoantibody Responses among the Disease and Control Groups

**Figure 3:**
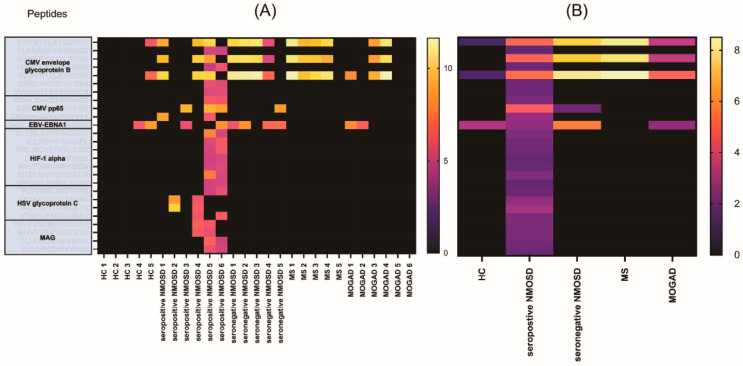
Heatmap of the statistically significant peptides. (**A**) Peptide IgG response per individual. (**B**) Average peptide IgG responses per group). Twenty-six statistically significant peptides selected from pairwise subgroup comparisons are shown as rows. Patients from the CNS inflammatory demyelinating disease subgroups and HC are shown as columns. The color indicates the peptide IgG response intensity (FU, VSN transformed, light yellow = higher response, black = lower response). A tendency for higher IgG responses in CMV-related peptides was observed in seronegative NMOSD and MS patients. IgG, immunoglobulin G; HC, healthy control group; MOGAD, myelin-oligodendrocyte glycoprotein antibody-associated disease; MS, multiple sclerosis; NMOSD, neuromyelitis optica spectrum disorder (NMOSD); CMV, cytomegalovirus; HSV, herpes simplex virus; MAG, myelin-associated glycoprotein; HIF, hypoxia-inducible factor.

The heatmap of 1252 pre-processed peptides, did not show apparent clustering in the HC and disease groups (Supplementary Figure S1). However, the peptides with statistical significance in pairwise comparisons showed apparent differences between the HC and other groups (MOGAD, MS, seronegative NMOSD, and seropositive NMOSD) (Figure 3). In particular, the IgG responses to CMV-related peptides were most distinguishable among the study groups (Figure 3).

## 4. Discussion

Peptide microarrays are a recently developed proteomic analysis method with a similar sensitivity to those of traditional methods [25]. Since various epitopes of viral antigens or autoantibodies often need to be analyzed simultaneously, this method is used to screen peptides related to autoimmune diseases [18,26,27]. The results of the present comprehensive peptide microarray screening of 2440 peptides selected based on previous literature [8] revealed CMV, HSV, MAG, HIF-1 alpha, and EBV-related peptides as possible candidates for discriminating CNS inflammatory demyelinating disorders.

Our results suggested that the CMV-related IgG autoantibody responses were most prominently observed in the NMOSD and MS groups. These IgG responses were the most discriminative in seronegative NMOSD and MS groups compared to the HC group. In addition, the types of CMV-related peptides were the highest in seropositive NMOSD (a total of seven peptides). These results suggest a possible link between a past CMV infection and the later occurrence of NMOSD and MS.

The specific pathogenic mechanism and correlation of CMV infection in MS and NMOSD has yet to be proven [28] and previous studies have shown inconsistent results [29,30,31]. Although CMV infection associated with a demyelinating form of Guillain-Barré syndrome, which is an autoimmune disorder in peripheral nervous system, has been reported [32,33], its impact on CNS inflammatory disorder is yet to be established. Recent studies showed that CMV seropositivity was negatively correlated with MS and that the low risk of MS was associated with CMV exposure in early life according to the hygiene hypothesis [29,30]. In contrast, the results of another study suggested that CMV might trigger an immunological mechanism in patients with MS by nonspecific B-cell hyperactivation [31]. In cases with NMOSD, only a few case reports have described a suspicious correlation for CMV infection before NMOSD development [34,35]. It should be noted that chronic latent infection is known to reach up to 70% of the general population, and this is one of the hurdles in CMV infection-related MS research [28]. Moreover, the prevalence of CMV seropositivity varies among ethnicities and socioeconomic statuses [36]. Therefore, further prospective studies with subjects stratified according to these factors are necessary to elucidate the role of CMV in the pathogenesis of CNS inflammatory demyelinating disorders in specific populations, and the results in this study should be interpreted with caution.

Other peptides related to EBV-EBNA1, HSV, HIF-1 alpha, and MAG also showed statistically significant relationships in this study. However, considering that the detection rate of EBV-EBNA1 antibody in healthy Korean children is up to 100%, IgG response to EBV-EBNA1 should be carefully considered [18,22,37,38,39]. In HSV, HIF-1, and MAG-related peptides, only small differences were observed between the disease and HC groups and the degrees of IgG responses were relatively low. Although HSV has been suggested to play a role in MS and NMOSD pathogenesis [40,41,42], clear evidence is lacking. The results of a Polish study suggested that only HHV-6 was correlated with MS, while HHV-1 and 2 were not [43]. More recently, MS and NMOSD showed no association with the anti-HSV-2 antibody [44]. HIF-1 alpha expression has been suggested to explain the hypoxic deep white matter lesions in CNS inflammatory demyelinating disorders. However, plausible evidence is lacking [15]. In addition, although the cerebrospinal fluid (CSF) production of MAG in MS was first reported in 1983 [45], no reliable reports have been suggested since that time.

The present study had several limitations, and its results should be interpreted with care. First, the small number of subjects did not allow the generation of a robust dataset with a reliable statistical outcome. Hence, further studies with larger sample sizes are required. However, we believe these data are valuable because no other study has compared the seropositivities of various peptides possibly related to CNS inflammatory demyelinating disorders, especially in the Asian population. Second, although we tried to exclude patients who underwent vigorous immunosuppressive therapy (e.g., plasma exchange, corticosteroids), previous oral immunosuppressive treatments still might have influenced our results [21,46,47]. Third, we performed only microarray analysis using a linear epitope without conformational testing, which was also described as a methodological limitation in previous literature [10]. The epitope sequence or degree of IgG response might differ in conformational studies.

In summary, we studied the differences in the IgG response to various peptides among MS, NMOSD, MOGAD, and HC groups using a peptide microarray. Relatively higher IgG responses to CMV-related peptides were observed in patients with MS and NMOSD, however the result should be interpreted with caution due to the seropositivity in the general population. Further detailed pathomechanistic research is needed to confirm these peptides as possible indicators for supportive differential diagnosis.

## Figures and Tables

**Table 1:** Clinical characteristics of the enrolled subjects.

	MS(*n* = 5)	Seropositive NMOSD (*n* = 6)	Seronegative NMOSD (*n* = 5)	MOGAD (*n* = 6)	HC (*n* = 5)
Male: Female (*n*:n)	2:3	1:5	1:4	2:4	3:2
Age, years (mean, range)	38.4 (26–47)	39.3 (24–50)	37.8 (27–52)	38.0 (29–53)	33.8 (26–45)
EDSS (median[quartile])	2.3 (2,3)	3 (2.25, 3.875)	3.5 (2.75, 3.5)	2.75 (1.625, 6.125)	-
Optic nerve lesion (*n*)	1	0	0	1	-
Spinal cord lesion (*n*)	1	2	0	0	-
Brain lesion (*n*)	2	0	0	0	-
Spinal cord with optic nerve lesion (*n*)	0	2	4	2	-
Spinal cord with brain lesion (*n*)	1	0	0	1	-
Optic nerve with brain lesion (*n*)	0	1	0	0	-

MS, multiple sclerosis; NMSOD, neuromyelitis optica spectrum disorder; MOGAD, anti-myelin oligodendrocyte glycoprotein-associated disease; HC, healthy control; EDSS, expanded disability status scale.

**Table 2:** Pairwise peptide IgG response comparisons of CMV-related peptides among disease and HC groups.

Comparison (A vs. B)	Antigen Type	Peptide	Mean Difference (A–B, FU)	*p* Values
MS vs. HC	CMV envelope glycoprotein B	GVNETIYNTTLKYGD	7.94	0.064 *
		NETIYNTTLKYGDVV	7.15	0.093 *
		ETIYNTTLKYGDVVG	6.9	0.098 *
Seronegative NMOSD vs. HC	CMV envelope glycoprotein B	GVNETIYNTTLKYGD	7.35	0.064 *
		NETIYNTTLKYGDVV	6.76	0.093 *
		ETIYNTTLKYGDVVG	6.28	0.098 *
Seropositive NMOSD vs. HC	CMV envelope glycoprotein B	TIRSEAEDSYHFSSA	1.81	0.088 *
		VVGVNTTKYPYRVCS	1.75	0.087 *
		LVAFLERADSVISWD	1.73	0.089 *
		FLERADSVISWDIQD	1.6	0.087 *
	CMV pp65	SDEELVTTERKTPRV	4.52	0.089 *
		TRQQNQWKEPDVYYT	2.45	0.092 *
		RGRLKAESTVAPEED	1.96	0.087 *
Seropositive NMOSD vs. MS	CMV envelope glycoprotein B	TIRSEAEDSYHFSSA	1.81	0.088 *
		VVGVNTTKYPYRVCS	1.75	0.087 *
		LVAFLERADSVISWD	1.73	0.089 *
		FLERADSVISWDIQD	1.6	0.087 *
	CMV pp65	SDEELVTTERKTPRV	4.52	0.089 *
		TRQQNQWKEPDVYYT	2.45	0.092 *
		RGRLKAESTVAPEED	1.96	0.087 *
Seropositive NMOSD vs. seronegative NMOSD	CMV envelope glycoprotein B	TIRSEAEDSYHFSSA	1.81	0.088 *
		VVGVNTTKYPYRVCS	1.75	0.087 *
		LVAFLERADSVISWD	1.73	0.089 *
		FLERADSVISWDIQD	1.6	0.087 *
	CMV pp65	TRQQNQWKEPDVYYT	2.45	0.092 *
		RGRLKAESTVAPEED	1.96	0.087 *
Seropositive NMOSD vs. MOGAD	CMV envelope glycoprotein B	TIRSEAEDSYHFSSA	1.81	0.088 *
		VVGVNTTKYPYRVCS	1.75	0.087 *
		LVAFLERADSVISWD	1.73	0.089 *
		FLERADSVISWDIQD	1.6	0.087 *
	CMV pp65	SDEELVTTERKTPRV	4.52	0.089 *
		TRQQNQWKEPDVYYT	2.45	0.092 *
		RGRLKAESTVAPEED	1.96	0.087 *

CMV, cytomegalovirus; MS, multiple sclerosis; HC, healthy control; FU, fluorescence unit; NMSOD, neuromyelitis optica spectrum disorder; MOGAD, anti-myelin oligodendrocyte glycoprotein-associated disease. A one-way analysis of variance (ANOVA) test with Benjamini–Hochberg false discovery rate (FDR) correction was performed. * *p* < 0.10.

## Data Availability

The data presented in this study are available on request from the corresponding author. The data are not publicly available due to restrictions, e.g., privacy or ethical.

## Supplementary Materials

The following are available online at https://www.mdpi.com/article/10.3390/diagnostics11081339/s1. Figure S1: Heatmap and hierarchical clustering of peptide microarray profiles using the Euclidean distance method. Table S1: Pairwise peptide IgG response comparisons of each disease group to the healthy control group. Table S2: Pairwise peptide IgG response comparisons between the MS and seropositive NMOSD groups. Table S3: Pairwise peptide IgG response comparisons between seropositive and seronegative NMOSD groups. Table S4: Pairwise peptide IgG response comparisons between seropositive NMOSD and MOGAD groups.
