# Supplementary material for: Serum Peptide Immunoglobulin G Autoantibody Response in Patients with Different Central Nervous System Inflammatory Demyelinating Disorders
Source: Diagnostics (Basel). 2021 Jul 26;11(8):1339. doi: 10.3390/diagnostics11081339 (PMC8392162; doi:10.3390/diagnostics11081339)
Supplement: Supplementary file 1 [file diagnostics-11-01339-s001.zip › diagnostics-1264176-supplementary.pdf]

Table S1. Pairwise peptide IgG response comparisons of each disease group to the healthy control group.

Comparison	Antigen type	Peptide	Mean difference (patient groups – HC, FU)	*p*-values
MS *vs*. HC	CMV envelope glycoprotein B	GVNETIYNTTLKYGD	7.94	0.064*
		NETIYNTTLKYGDVV	7.15	0.093*
		ETIYNTTLKYGDVVG	6.90	0.098*
Seronegative NMOSD *vs*. HC	CMV envelope glycoprotein B	GVNETIYNTTLKYGD	7.35	0.064*
		NETIYNTTLKYGDVV	6.76	0.093*
		ETIYNTTLKYGDVVG	6.28	0.098*
Seropositive NMOSD *vs*. HC	CMV pp65	SDEELVTTERKTPRV	4.52	0.089*
	HSV glycoprotein C	TASTGPTITAGAVTN	2.73	0.095*
	CMV pp65	TRQQNQWKEPDVYYT	2.45	0.092*
	HSV glycoprotein C	SETASTGPTITAGAV	2.40	0.092*
	HIF-1 alpha	DLEMLAPYIPMDDDF	2.11	0.094*

	HIF-1 alpha	RITELMGYEPEELLG	2.07	0.092*
	MAG	TEVEVSCMVPDNCPE	2.03	0.088*
	HSV glycoprotein C	VTGPLPTQRLIIGEV	2.02	0.087*
	CMV pp65	RGRLKAESTVAPEED	1.96	0.087*
	MAG	NIVVPPEVVAGTEVE	1.95	0.088*
	MAG	TLTEELAEYAEIRVK	1.87	0.091*
	HIF-1 alpha	ICEPIPHPSNIEIPL	1.86	0.088*
	CMV envelope glycoprotein B	TIRSEAEDSYHFSSA	1.81	0.088*
	HIP-1 alpha	ITELMGYEPEELLGR	1.81	0.089*
	MAG	VLDIVNTPNIVVPPE	1.76	0.088*
	CMV envelope glycoprotein B	VVGVNTTKYPYRVCS	1.75	0.087*
	HSV glycoprotein C	PLPTQRLIIGEVTPA	1.73	0.088*

	CMV envelope glycoprotein B	LVAFLERADSVISWD	1.73	0.089*
	HIF-1 alpha	MVLTDDGDMIYISDN	1.67	0.088*
	HIF-1 alpha	VPEEELNPKILALQN	1.63	0.087*
	HIF-1 alpha	LVLICEPIPHPSNIE	1.63	0.087*
	CMV envelope glycoprotein B	FLERADSVISWDIQD	1.60	0.087*

CMV, cytomegalovirus; MS, multiple sclerosis; HC, healthy control; NMSOD, neuromyelitis optica spectrum disorder; HSV, herpes simplex virus; MAG, myelin-associated glycoprotein; HIF, hypoxia-inducible factor. A one-way analysis of variance (ANOVA) test with Benjamini–Hochberg false discovery rate (FDR) correction was performed. *$p < 0.10$.

Table S2. Pairwise peptide IgG response comparisons between the MS and seropositive NMOSD groups.

Antigen type	Peptide	Mean difference (seropositive NMOSD – MS, FU)	*p*-values
CMV pp65	SDEELVTTERKTPRV	4.52	0.089*
HSV glycoprotein C	TASTGPTITAGAVTN	2.73	0.095*
CMV pp65	TRQQNQWKEPDVYYT	2.45	0.092*
HSV glycoprotein C	SETASTGPTITAGAV	2.40	0.092*
HIF-1 alpha	DLEMLAPYIPMDDDF	2.11	0.094*
HIF-1 alpha	RITELMGYEPEELLG	2.07	0.092*
MAG	TEVEVSCMVPDNCPE	2.03	0.088*
HSV glycoprotein C	VTGPLPTQRLIIGEV	2.02	0.087*
CMV pp65	RGRLKAESTVAPEED	1.96	0.087*
MAG	NIVVPPEVVAGTEVE	1.95	0.088*
MAG	TLTEELAEYAEIRVK	1.87	0.091*

HIF-1 alpha	ICEPIPHPSNIEIPL	1.86	0.088*
CMV envelope glycoprotein B	TIRSEAEDSYHFSSA	1.81	0.088*
HIF-1 alpha	ITELMGYEPEELLGR	1.81	0.089*
MAG	VLDIVNTPNIVVPPE	1.76	0.088*
CMV envelope glycoprotein B	VVGVNTTKYPYRVCS	1.75	0.087*
HSV glycoprotein C	PLPTQRLIIGEVTPA	1.73	0.088*
CMV envelope glycoprotein B	LVAFLERADSVISWD	1.73	0.089*
HIF-1 alpha	MVLTDDGDMIYISDN	1.67	0.088*
HIF-1 alpha	VPEEELNPKILALQN	1.63	0.087*
HIF-1 alpha	LVLICEPIPHPSNIE	1.63	0.087*
CMV envelope glycoprotein B	FLERADSVISWDIQD	1.60	0.087*

IgG, immunoglobulin G; MS, multiple sclerosis; NMSOD, neuromyelitis optica spectrum disorder; CMV, cytomegalovirus; HSV, herpes simplex virus; MAG, myelin-associated glycoprotein; HIF, hypoxia-inducible factor. A one-way analysis of variance (ANOVA) test with Benjamini–Hochberg false discovery rate (FDR) correction was performed. *$p < 0.10$.

Table S3. Pairwise peptide IgG response comparisons between seropositive and seronegative NMOSD groups.

Antigen	peptide	Mean difference (seronegative NMOSD – seropositive NMOSD, FU)	*p*-values
CMV envelope glycoprotein B	FLERADSVISWDIQD	-1.60	0.087*
HIF-1 alpha	LVLICEPIPHPSNIE	-1.63	0.087*
HIF-1 alpha	VPEEELNPKLALQN	-1.63	0.087*
HIF-1 alpha	MVLTDDGDMIYISDN	-1.67	0.088*
CMV envelope glycoprotein B	LVAFLERADSVISWD	-1.73	0.089*
HSV glycoprotein C	PLPTQRLIIGEVTPA	-1.73	0.088*
CMV envelope glycoprotein B	VVGVNTTKYPYRVCS	-1.75	0.087*
MAG	VLDIVNTPNIVVPPE	-1.76	0.088*
HIF-1 alpha	ITELMGYEPEELLGR	-1.81	0.089*
CMV envelope glycoprotein B	TIRSEAEDSYHFSSA	-1.81	0.088*
HIF-1 alpha	ICEPIPHPSNIEIPL	-1.86	0.088*

MAG	TLTEELAEYAEIRVK	-1.87	0.091*
MAG	NIVVPPEVVAGTEVE	-1.95	0.088*
CMV pp65	RGRLKAESTVAPEED	-1.96	0.087*
HSV glycoprotein C	VTGPLPTQRLIIGEV	-2.02	0.087*
MAG	TEVEVSCMVPDNCPE	-2.03	0.088*
HIF-1 alpha	RITELMGYEPEELLG	-2.07	0.092*
HIF-1 alpha	DLEMLAPYIPMDDDF	-2.11	0.094*
HSV glycoprotein C	SETASTGPTITAGAV	-2.40	0.092*
CMV pp65	TRQQNQWKEPDVYYT	-2.45	0.092*
HSV glycoprotein C	TASTGPTITAGAVTN	-2.73	0.095*

IgG, immunoglobulin G; MS, multiple sclerosis; NMSOD, neuromyelitis optica spectrum disorder; CMV, cytomegalovirus; HSV, herpes simplex virus; MAG, myelin-associated glycoprotein; HIF, hypoxia-inducible factor. A one-way analysis of variance (ANOVA) test with Benjamini–Hochberg false discovery rate (FDR) correction was performed. *$p < 0.10$.

Table S4. Pairwise peptide IgG response comparisons between seropositive NMOSD and MOGAD groups.

Antigen	Peptide	Mean difference (MOGAD-seropositive NMOSD, FU)	*p*-value
CMV envelope glycoprotein B	FLERADSVISWDIQD	-1.60	0.087*
HIF-1 alpha	LVLICEPIPHPSNIE	-1.63	0.087*
HIF-1 alpha	VPEEELNPKILALQN	-1.63	0.087*
HIF-1 alpha	MVLTDDGDMIYISDN	-1.67	0.088*
CMV envelope glycoprotein B	LVAFLERADSVISWD	-1.73	0.089*
HSV glycoprotein C	PLPTQRLIIGEVTPA	-1.73	0.080*
CMV envelope glycoprotein B	VVGVNTTKYPYRVCS	-1.75	0.087*
MAG	VLDIVNTPNIVVPPE	-1.76	0.088*
HIF-1 alpha	ITELMGYEPEELLGR	-1.81	0.089*
CMV envelope glycoprotein B	TIRSEAEDSYHFSSA	-1.81	0.088*
HIF-1 alpha	ICEPIPHPSNIEIPL	-1.86	0.088*

MAG	TLTEELAEYAEIRVK	-1.87	0.091*
MAG	NIVVPPEVVAGTEVE	-1.95	0.088*
CMV pp65	RGRLKAESTVAPEED	-1.96	0.087*
HSV glycoprotein C	VTGPLPTQRLIIGEV	-2.02	0.087*
MAG	TEVEVSCMVPDNCPE	-2.03	0.088*
HIF-1 alpha	RITELMGYEPEELLG	-2.07	0.092*
HIF-1 alpha	DLEMLAPYIPMDDDF	-2.11	0.094*
HSV glycoprotein C	SETASTGPTITAGAV	-2.40	0.092*
CMV pp65	TRQQNQWKEPDVYYT	-2.45	0.092*
HSV glycoprotein C	TASTGPTITAGAVTN	-2.73	0.095*
CMV pp65	SDEELVTTERKTPRV	-4.52	0.089*

IgG, immunoglobulin G; MS, multiple sclerosis; NMSOD, neuromyelitis optica spectrum disorder; CMV, cytomegalovirus; HSV, herpes simplex virus; MAG, myelin-associated glycoprotein; HIF, hypoxia-inducible factor A one-way analysis of variance (ANOVA) test with Benjamini–Hochberg false discovery rate (FDR) correction was performed. *$p < 0.10$.

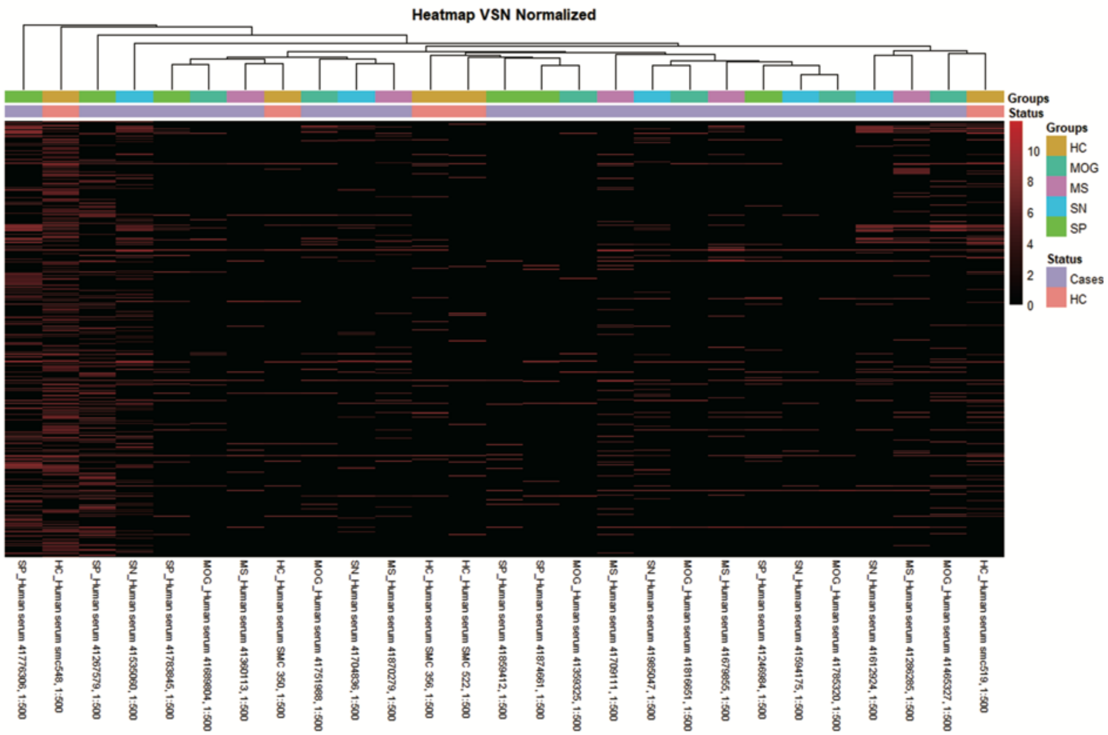

Figure S1. Heatmap and hierarchical clustering of peptide microarray profiles using the Euclidean distance method. The HC and patient groups show no clear clustering. SP, seropositive; HC, healthy control; MOG, myelin-oligodendrocyte glycoprotein antibody-associated disease; MS, multiple sclerosis; SP, seropositive. The sera were diluted to 1: 500.
